# Evaluating the Relationship between Cathepsins and Papillary Thyroid Carcinoma: A Mendelian Randomization Study

**DOI:** 10.2174/0118715303305715240912172648

**Published:** 2025-01-08

**Authors:** Liu Muge, Xiao Xiongsheng, Jin Ling, Li Siyi, Zheng Changwei, Chen Zhengde, Chen Zhuoting, Zhang Zhi

**Affiliations:** 1 Department of Vascular and Thyroid Surgery, Affiliated Hospital of Guangdong Medical University, Guangdong, China;; 2 Department of Breast Surgery, Affiliated Hospital of Guangdong Medical University, Guangdong, China

**Keywords:** Papillary thyroid carcinoma, cathepsins, mendelian randomization, causal relationships, lysosomal proteases, fineneedle aspiration

## Abstract

**Background:**

Papillary Thyroid Carcinoma (PTC) is the most common thyroid cancer, with an etiology and progression that are not fully understood. Research suggests a link between cathepsins and PTC, but the causal nature of this link is unclear. This study uses Mendelian Randomization (MR) to investigate if cathepsins causally influence PTC risk.

**Methods:**

We applied univariable and multivariable MR analyses using genetic variants as proxies for cathepsin levels. Genetic data for cathepsins were sourced from the INTERVAL study, while PTC data came from the Finnish Genome-Wide Association Study database. Our analysis employed several MR methods, including the Inverse Variance Weighted (IVW) approach, MR-Egger, and the Weighted Median method, to provide comprehensive insights and address possible pleiotropy.

**Results:**

MR findings suggest a significant causal association between higher cathepsin levels and increased PTC risk. Notably, genetic variants indicating higher cathepsin Z expression were positively causal associated with PTC risk (OR:1.1190, 95% CI: 1.0029-1.2486), multivariable analysis confirmed significant carcinogenesis role of cathepsin Z in PTC (OR: 1.1593, 95% CI: 1.0137-1.3258), with results consistent across various tests, indicating a robust relationship.

**Conclusion:**

This study established a causal link between cathepsin levels and PTC risk, emphasizing the roles of cathepsin Z in its progression. These insights could lead to new therapeutic strategies targeting these enzymes. Further research is necessary to understand the underlying biological mechanisms and their clinical implications.

## 1 INTRODUCTION

Papillary Thyroid Carcinoma (PTC), the most common thyroid malignancy, constitutes a major portion of global endocrine cancers [1]. Despite its prevalence, the complex etiology of PTC remains incompletely understood, necessitating further investigation into its molecular mechanisms. Advances in genomic medicine have highlighted potential genetic and molecular contributors to PTC, with cathepsins emerging as key molecules of interest [2-5].

Cathepsins, a group of lysosomal proteases, are primarily known for protein degradation but are increasingly implicated in pathological processes, including cancers [6-10]. While overexpression of cathepsins has been linked to various cancers, their specific role in papillary thyroid carcinoma (PTC) remains unclear, highlighting a critical area for further research. Genetic testing is a critical tool for diagnosing thyroid cancer and complements fine-needle aspiration (FNA) results. The expression of protease genes may enhance diagnostic accuracy. However, the diagnostic clarity of Bethesda categories III and IV from thyroid nodule FNA remains limited, and there is a risk of malignancy even in category II results. Research by Professor Francesk Mulita and Ioannis Maroulis found that 1.53% (8/522) of patients with Bethesda II results were later confirmed to have malignant tumors, predominantly papillary thyroid carcinomas [11]. Additionally, 19.19% (66/344) of patients with Bethesda III results were diagnosed with malignancies [12]. Therefore, more precise genetic diagnostics could significantly improve patient outcomes.

Our study aims to address this knowledge gap using Mendelian Randomization (MR) [13, 14], a method that leverages genetic variants as instruments to infer causal relationships between modifiable exposures and health outcomes, in this case, the association between cathepsin levels and the risk of developing papillary thyroid carcinoma (PTC), as well as the reverse of it. MR is particularly effective in assessing causality while mitigating confounding factors and reverse causation, which are common limitations in observational studies.

By investigating genetic variants associated with cathepsin expression, we aim to establish a potential causal link between cathepsin levels and the incidence of papillary thyroid carcinoma (PTC). This study not only deepens our understanding of PTC's molecular mechanisms but also informs the development of targeted therapies. A thorough examination of the role of cathepsins in PTC could lead to innovative management and treatment strategies. Ultimately, this research seeks to significantly advance the current knowledge of PTC and improve our capacity to address this complex disease.

## 2 MATERIALS AND METHODS

### 2.1 Study Design

MR, a cutting-edge approach in genetic epidemiology, utilizes genetic variations as tools (instrumental variables) to infer cause-and-effect relationships between alterable risk elements and subsequent health outcomes [15, 16]. This method employs the random inheritance of genes from parents to offspring during the formation of gametes, thus mirroring the random assignment typical in controlled experiments. MR plays a crucial role in diminishing the impact of confounders and the issue of reverse causality, which are common in observational studies, thereby providing a more solid and dependable determination of causative impacts. It requires the use of credible genetic tools that demonstrate a pronounced link with the risk factor but not the health outcome.

We analyzed the cause-and-effect relationships of 9 cathepsins on PTC and the reverse interaction through bi-directional, single-variant Mendelian randomization studies and comprehensive Mendelian randomization evaluations [17]. MR derives instrumental variables (IV) from single nucleotide polymorphism data to signify the risk factor. Effective IVs must adhere to three criteria:

- Instrumental variables should be closely associated with the risk factor;
- Instrumental variables should not be linked to any other confounding elements affecting the outcome;
- Instrumental variables should not have a direct connection to the outcome.

### 2.2 GWAS Data Sources for Cathepsins and PTC

Genome-Wide Association Study (GWAS) Data for various cathepsin levels, derived from the INTERVAL study involving 3301 European individuals [18], was employed. We utilized the eleventh release (R11version) of the FinnGen project database for the GWAS data on papillary thyroid carcinoma, including 346859 Finnish individuals. The FinnGen study is a large-scale genomics initiative that has analyzed over 500,000 biological samples from Finnish biobanks, correlating genetic variations with health data to elucidate disease mechanisms and susceptibilities. This project represents a collaboration among research organizations and biobanks within Finland as well as international industry partners [19].

### 2.3 Selection of Instrumental Variables

The selection of cathepsin-related instrumental variables followed specific criteria: (a) linkage disequilibrium (LD) r2 value among tools being under 0.001 within a 10,000 kb span; (b) defining significance thresholds, ensuring *p*-values are below the genome-wide significance level established in the study (**5** × **10**^−**6**, this value was determined based on the constraints of the sample size). The instrumental variables for PTC as risk factors followed similar guidelines, with the exception of the significance thresholds set at **5** × **10**^−**8**. The effectiveness of the independent variables was assessed by computing R-squared and F statistics, and any independent variables with F values less than 10 were omitted.

### 2.4 Statistical Analysis

All procedures were conducted using R (version 4.3.1, RRID: SCR_001905). Methods like inverse variance weighting, MR-Egger, and weighted median were employed utilizing the TwoSampleMR package (version 0.5.7, RRID: SCR_019010) and the Mendelian Randomization package (version 0.9.0, RRID: None). Heterogeneity assessments were carried out using Cochran’s Q test. For evaluating horizontal pleiotropy, both MR-Egger and MR-PRESSO methods were employed. To ascertain the solidity and variability of the results, leave-one-out sensitivity analysis, scatter diagrams, and funnel charts were employed.

## 3 RESULTS

In this comprehensive study, we rigorously analyzed the relationship between various cathepsins and the risk of papillary thyroid carcinoma (PTC), mainly using the Inverse Variance Weighted method of Mendelian Randomization. We meticulously evaluated the potential causal links for several cathepsins, including B, E, F, G, H, O, S, L2, and Z. Our extensive analysis, based on the odds ratios (ORs) and confidence intervals (CIs), revealed that for the majority of these cathepsins, there was no significant causal association with the risk of PTC. The ORs and CIs of these cathepsins, except Z, did not indicate a robust causal relationship, suggesting that these specific cathepsins may not play a significant role in the etiology of PTC.

However, our findings identified a notable exception with cathepsin Z. We observed a statistically significant association, with an OR of 1.1190 and a 95% CI ranging from 1.0029 to 1.2486 (*P* =0.0442). This significant correlation suggests a potential causal link between elevated levels of cathepsin Z and an increased risk of developing PTC, as presented in Table **1**. This intriguing finding underscores the importance of cathepsin Z in the pathogenesis of PTC.

Additionally, we examined the expression levels of various cathepsins in patients diagnosed with PTC. The IVW results for these cathepsins indicated no significant expression change in PTC patients, as detailed in Table (**2**).

**Fig. (1):**
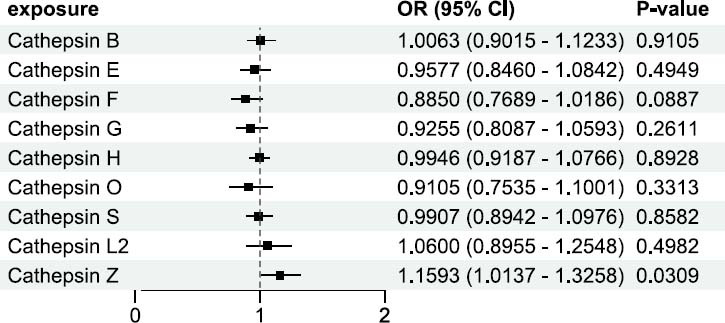
The results of multivariable MR analysis of cathepsins on PTC. By multivariable MR analysis, cathepsin Z still showed a positive causal relationship with PTC, consistent with the result of the univariable MR analysis.

To address the complex interplay and tight expression correlation among different cathepsins, we also conducted a multivariable Mendelian Randomization analysis. This multivariable approach was essential in discerning the specific contributions of each cathepsin to PTC risk. In this analysis, most cathepsins (except Z) did not exhibit a significant causal relationship with PTC risk. The ORs for these cathepsins hovered narrowly around “1”, accompanied by wide CIs and high *p*-values, which collectively suggested a negligible causal influence. However, the results were markedly different for cathepsin Z. It demonstrated a more pronounced OR of 1.1593 (95% CI: 1.0137-1.3258) with a *p*-value of 0.0309 (Fig. **1**). This finding pointed to a potentially significant causal relationship between cathepsin Z and an increased risk of PTC. It suggested a specific biological pathway involving cathepsin Z that merited deeper investigation and could hold key insights into the pathogenesis of PTC.

**Fig. (2):**
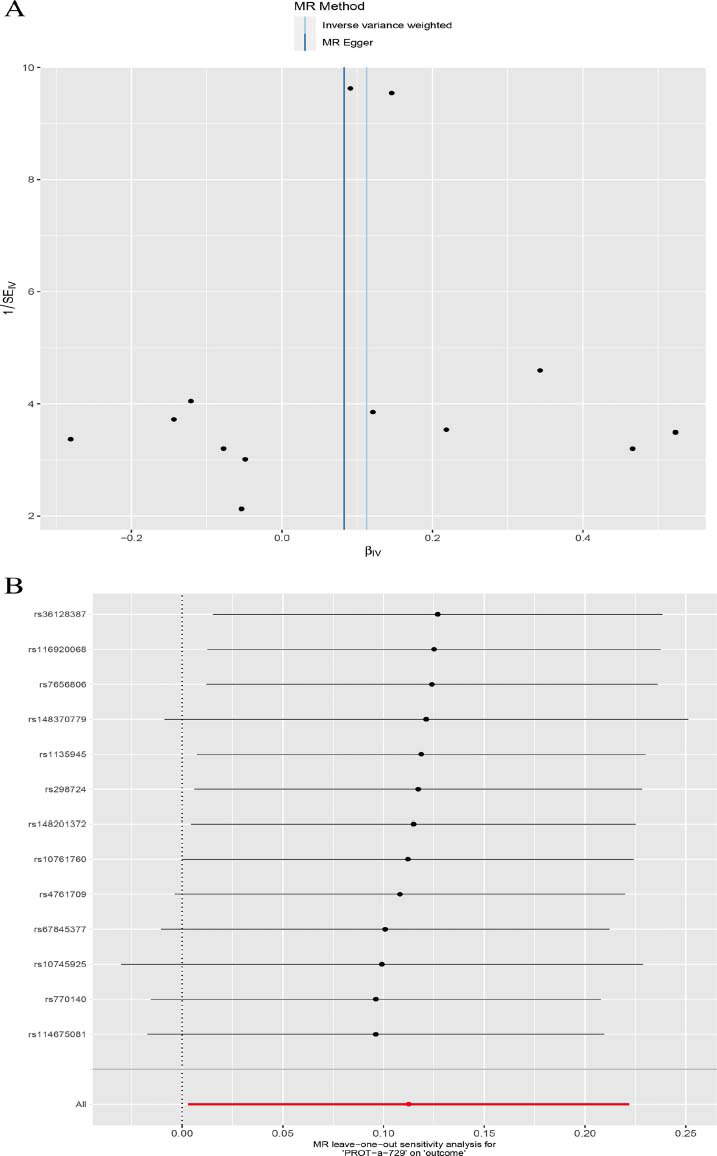
The funnel plot and sensitivity analysis of the causal associations between cathepsin Z and PTC. The results showed our analysis was robust. (**A**): The univariable funnel plot of cathepsin Z on PTC; (**B**): The univariable sensitivity analysis of cathepsin Z on PTC.

Furthermore, we employed the MR-Egger and MR-PRESSO approaches to ascertain the presence of horizontal pleiotropy (Table **3**). The outcomes were also subjected to sensitivity analyses, and the stability and reliability of these findings were elucidated through the funnel plot and sensitivity analysis (Fig. **2**).

## 4 DISCUSSION

In this in-depth discussion, we scrutinize the intricate findings of our study regarding the associations between cathepsins and papillary thyroid carcinoma (PTC), utilizing the Inverse Variance Weighted (IVW) method in Mendelian Randomization. The lack of a strong causal link for most cathepsins, including B, E, F, G, H, O, S, and L2, suggests a minimal individual contribution to PTC pathogenesis. This observation aligns with the multifactorial nature of PTC [20-22], where a complex interplay of genetic and environmental factors is at play, and no single genetic determinant may dominate the etiology.

The potential relationship between cathepsins and tumors has been validated in various cancers, and the majority of studies indicate that elevated expression of cathepsins is associated with poor prognosis. In the investigative research, Anastasia S. Frolova and her team examined the expression, positioning, and development of eleven cysteine cathepsins across embryonic kidney HEK 293 cells and renal carcinoma cell lines (769-P and A-498). The findings revealed that the expression levels of cathepsins V, B, Z, L, and S in renal cancer cells were 3 to 9 times greater than in embryonic cells. The cysteine cathepsins were situated within the cell nucleus, and the mature versions of these enzymes were more commonly observed in cancer cells than in embryonic cells [23]. Song Wei and associates identified that the concentrations of cathepsin F (CTSF) and Fibulin-1 are elevated in the serum and tissue of non-small cell lung cancer (NSCLC) patients with brain metastases, in contrast to patients without brain metastases or individuals with primary brain tumors. Simultaneously, alterations in serum CTSF concentrations can indicate the treatment outcomes of NSCLC patients with brain metastases. Furthermore, increased expression of CTSF in the tissues of these patients correlates with shorter progression-free survival [24]. Fan Zhang found that concentrations of CTSZ were linked to the outcomes of individuals with clear cell renal cell carcinoma (ccRCC) (hazard ratio = 1.5, *P* = 0.007), where elevated CTSZ levels corresponded to worse prognoses during anti-PD-1 monotherapy. Single-cell RNA sequencing data indicated that CTSZ was uniquely expressed in macrophages within ccRCC [25].

In studies related to thyroid cancer, the role of cathepsins in its development and progression is not yet fully understood; particularly, the functions of cathepsin Z in papillary thyroid carcinoma have not been definitively determined. In Nthy-ori 3-1 cell cultures, Alaa Al-Hashimi and their team noted that cells expressing N-terminally truncated cathepsin V-eGFP demonstrated increased proliferation compared to wild-type counterparts, indicating its influence on enhanced cell growth [26]. In their study, Juan Tan and associates found that the expression of Cathepsin S correlated with lymph node metastasis and adverse outcomes in papillary thyroid carcinoma. Employing comprehensive bioinformatics analysis, they suggested that Cathepsin S was an independent risk factor for poor disease-free survival and that high expression of Cathepsin S was independently linked with lymph node metastasis [4]. Eun-Kyung Kim and fellow researchers utilized mass spectrometry to investigate the secretome of typical thyroid cells, papillary thyroid carcinoma (PTC) cells, and anaplastic thyroid cancer cells, with the goal of pinpointing distinctive markers for thyroid carcinoma. They noted the most elevated expression levels of Cathepsin B (CTSB) in PTC cells, observing that CTSB concentrations in cancerous samples surpassed those in healthy tissue. Moreover, in individuals with thyroid cancer, heightened levels of CTSB were positively correlated with lymph node metastasis (LNM) and advanced N staging, suggesting its possible involvement in the advancement and metastatic dissemination of the condition [5]. Yaqiong Wang and colleagues conducted a risk analysis study in a family where all six siblings were predisposed to hereditary thyroid cancer. Through sequencing analysis of blood samples, the findings revealed that five of the six family members carried a candidate gene mutation in CTSF. Additionally, immunohistochemical analysis of thyroid cancer tissue samples indicated that the expression of CTSF in patients with the mutation was higher compared to those with the wild-type gene and the surrounding non-cancerous tissue [2].

There is a paucity of research on the intrinsic relationship between cathepsin Z and papillary thyroid carcinoma. The standout finding of cathepsin Z in our results, showing a significant correlation with PTC risk, pivots our discussion to its potential unique role in thyroid carcinogenesis. The mechanisms by which cathepsin Z might influence PTC are multifaceted. Its involvement in tissue remodeling [27, 28], a key aspect of cancer development, is particularly notable. The increased levels of cathepsin Z could signify a response to tumorigenic stimuli or an aberration in the cellular microenvironment of the thyroid gland, leading to unchecked growth and metastasis. The critical angle at which cathepsin Z could exert its influence on PTC might be its role in degrading extracellular matrix components, which might enhance tumor invasiveness [29-31]. Additionally, its functionality in cleaving specific substrates might impact signaling pathways pivotal for cell proliferation and survival. These hypotheses necessitate further exploration to elucidate the exact biological mechanisms at play.

The comprehensive multivariable MR analysis, accounting for correlations among different cathepsins, highlights the necessity of considering the collective interplay of proteases in cancer development. The absence of significant causal links for most cathepsins in this multivariable context suggests either their roles are overshadowed by more potent factors or their effects are highly contingent on specific cellular contexts. Cathepsin Z still stably shows a significant correlation with PTC risk, enhancing the credibility of its effect.

We turn our attention to the broader clinical and research implications. The prospect of utilizing cathepsin Z as a biomarker for PTC risk assessment or as a therapeutic target is particularly exciting. This could herald a new era in the personalized management of PTC, potentially extending to other cancers where cathepsins play a role. This study opens avenues for future research, particularly in personalized medicine approaches for PTC management. Understanding the intricate roles of different cathepsins could lead to more targeted and effective therapeutic strategies, enhancing patient outcomes. The insights garnered here lay the groundwork for such endeavors, highlighting the significance of genetic studies in unraveling the complex molecular landscapes of cancer. Our study exhibited several limitations, including suboptimal statistical power and a deficiency in the diversity of GWAS data sources. However, we believe that emphasizing the need for functional studies is paramount. Such investigations are vital to validate our epidemiological findings and offer concrete biological insights into the roles of cathepsins, particularly Z, in the pathogenesis of PTC.

## CONCLUSION

In conclusion, our study utilizing Mendelian Randomization suggests a specific association between cathepsin levels (Z) and the risk or progression of PTC, while no significant causal links were found for other cathepsins. The results advocate for a deeper exploration into the mechanistic roles of cathepsin Z in the occurrence and development of PTC. These insights reinforce the potential of genetic and proteomic profiling in understanding cancer etiology and highlight avenues for future research, which may include targeted therapies and improved diagnostic strategies for PTC.

## Figures and Tables

**Table 1:** The results of univariable MR analysis of cathepsins on PTC.

**Cathepsin**	**Method**	**Nsnp**	**OR**	**OR (95% CI)**	***P*-value**
Cathepsin B	MR-Egger	20	0.8737	(0.6560-1.1637)	0.3682
	Weighted median	20	1.0007	(0.8516-1.1760)	0.9931
	IVW	20	0.9850	(0.8728-1.1116)	0.8063
Cathepsin E	MR-Egger	9	0.9904	(0.7946-1.2344)	0.9338
	Weighted median	9	0.9683	(0.8085-1.1597)	0.7265
	IVW	9	0.9377	(0.8217-1.0701)	0.3399
Cathepsin F	MR-Egger	12	0.9080	(0.6797-1.2129)	0.5282
	Weighted median	12	0.9335	(0.7850-1.1101)	0.4363
	IVW	12	0.9974	(0.8811-1.1291)	0.9676
Cathepsin G	MR-Egger	12	0.7050	(0.5193-0.9572)	0.0490
	Weighted median	12	0.8078	(0.6706-0.9731)	0.0247
	IVW	12	0.8920	(0.7745-1.0273)	0.1126
Cathepsin H	MR-Egger	11	0.9793	(0.8606-1.1145)	0.7589
	Weighted median	11	0.9668	(0.8871-1.0537)	0.4424
	IVW	11	1.0082	(0.9210-1.1036)	0.8599
Cathepsin O	MR-Egger	12	1.0464	(0.7314-1.4972)	0.8089
	Weighted median	12	0.8722	(0.7062-1.0771)	0.2041
	IVW	12	0.9139	(0.7822-1.0677)	0.2566
Cathepsin S	MR-Egger	23	1.0555	(0.8921-1.2488)	0.5356
	Weighted median	23	1.0697	(0.9288-1.2321)	0.3497
	IVW	23	0.9997	(0.9060-1.1030)	0.9945
Cathepsin L2	MR-Egger	12	0.9606	(0.6122-1.5071)	0.8645
	Weighted median	12	0.9506	(0.7524-1.2010)	0.6710
	IVW	12	1.0385	(0.8737-1.2345)	0.6682
Cathepsin Z	MR-Egger	13	1.0860	(0.9128-1.2920)	0.3720
	Weighted median	13	1.1236	(0.9765-1.2928)	0.1036
	IVW	13	1.1190	(1.0029-1.2486)	0.0442*

**Note:** Table **1** presents that cathepsin Z as an exposure had a positive causal relationship with PTC by IVW method of MR analysis, compared with other cathepsins.

*P Value

**Table 2:** The results of univariable MR analysis of PTC on cathepsins.

**Cathepsin**	**Method**	**Nsnp**	**OR**	**OR (95% CI)**	***P*-value**
Cathepsin B	MR-Egger	8	1.0335	(0.8627-1.2381)	0.7328
	Weighted median	8	1.0342	(0.9549-1.1201)	0.4090
	IVW	8	1.0151	(0.9517-1.0828)	0.6487
Cathepsin E	MR-Egger	8	1.0299	(0.8597-1.2337)	0.7604
	Weighted median	8	0.9795	(0.9065-1.0584)	0.6004
	IVW	8	0.9394	(0.8807-1.0020)	0.0577
Cathepsin F	MR-Egger	8	0.9494	(0.7926-1.1373)	0.5938
	Weighted median	8	0.9555	(0.8769-1.0410)	0.2977
	IVW	8	0.9388	(0.8801-1.0013)	0.0550
Cathepsin G	MR-Egger	8	1.0692	(0.8419-1.3578)	0.6031
	Weighted median	8	1.0637	(0.9735-1.1623)	0.1717
	IVW	8	1.0271	(0.9482-1.1125)	0.5119
Cathepsin H	MR-Egger	8	0.8344	(0.6942-1.0031)	0.1022
	Weighted median	8	0.9864	(0.9073-1.0726)	0.7492
	IVW	8	0.9918	(0.9173-1.0725)	0.8372
Cathepsin O	MR-Egger	8	0.8855	(0.7314-1.0720)	0.2588
	Weighted median	8	1.0007	(0.9162-1.0929)	0.9882
	IVW	8	1.0025	(0.9325-1.0777)	0.9461
Cathepsin S	MR-Egger	8	1.0920	(0.8527-1.3985)	0.5117
	Weighted median	8	1.0670	(0.9754-1.1673)	0.1568
	IVW	8	1.0407	(0.9578-1.1307)	0.3460
Cathepsin L2	MR-Egger	8	0.8711	(0.7272-1.0435)	0.1847
	Weighted median	8	0.9292	(0.8552-1.0097)	0.0832
	IVW	8	0.9615	(0.9014-1.0256)	0.2330
Cathepsin Z	MR-Egger	8	1.0429	(0.8705-1.2493)	0.6647
	Weighted median	8	1.0122	(0.9305-1.1010)	0.7777
	IVW	8	1.0214	(0.9576-1.0895)	0.5199

**Note:** Table **2** presents that PTC as an exposure had no causal relationship with cathepsins by the IVW method of MR analysis.

**Table 3:** The pleiotropy test of MR analysis.

**Method**	**Exposure**	**Outcome**	***P*-value**
Univariable MR analysis	Cathepsin Z	PTC	0.6714
Multivariable MR analysis	Cathepsin Z	PTC	0.7470

**Note:** The pleiotropy test indicated that there was no pleiotropy in the MR analysis.

## Data Availability

The raw data supporting the conclusions of this paper can be accessed in public databases: https://gwas.mrcieu.ac.uk and https://www.finngen.fi/en/access_results.

## LIST OF ABBREVIATIONS

CIs: Confidence Intervals
GWAS: Genome-Wide Association Study
IV: Instrumental Variables
LNM: Lymph Node Metastasis
MR: Mendelian Randomization
ORs: Odds Ratios
PTC: Papillary Thyroid Carcinoma
